# All‐Arthroscopic Trochlear Osteochondral Scaffold Matrix Implantation for Osteochondral Defects of the Femoral Trochlea

**DOI:** 10.1002/atn2.70306

**Published:** 2026-10-08

**Authors:** Matthew Varacallo, Jared Ham

**Affiliations:** ^1^ Penn Highlands DuBois DuBois Pennsylvania U.S.A.

## Abstract

The treatment of osteochondral defects via an aragonite‐based osteochondral scaffold has been established via mini‐arthrotomy. This report describes a variation of the established procedure to perform and complete the scaffold implantation in the femoral trochlea via a reproducible, all‐arthroscopic technique. Emphasis is placed on patient selection, portal planning, perpendicular socket preparation, syringe‐based implant delivery, and recessed press‐fit fixation. Technical pearls and pitfalls specific to the patellofemoral joint are highlighted to facilitate safe and accurate execution.

## Graphical abstract

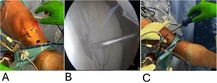

**VIDEO 1:** The all‐arthroscopic trochlear osteochondral scaffold matrix implantation for osteochondral defects of the femoral trochlea. This video describes a single‐stage, all‐arthroscopic approach to trochlear osteochondral scaffold matrix implantation. The video describes trochlear lesion assessment, portal preparation, and assessment for use of the CartiHeal Agili‐C tools and implant (Smith & Nephew, Andover, MA). The video details the use of the specialized tools to insert the guidewire and then drill, shape, clean, and bevel the socket for implantation using a modified 3‐mL syringe. The video is captured through the anterolateral portal with all tools, devices, and implants being placed through the anteromedial portal to provide a successful implant placement in the right knee of the patient. Video content can be viewed at https://doi.org/10.1002/atn2.70306.

Focal osteochondral defects of the femoral trochlea present a technically demanding subset of cartilage pathology because of high patellofemoral contact pressures, shear forces, and the requirement for precise implant orientation.^1^ Many established surgical options involve staged procedures, donor‐site morbidity, or delayed implantation; however, osteochondral allograft transplantation introduces logistical challenges related to graft availability, sizing, and storage.^2^, ^3^, ^4^, ^5^, ^6^, ^7^ These techniques often require open exposure to achieve perpendicular access, potentially increasing morbidity and limiting future treatment options.^2^, ^8^, ^9^ Arthroscopic implantation preserves future treatment options while maintaining a biologically favorable environment for osteochondral restoration, although not all implants or techniques are suitable for this approach.^10^

The biphasic osteochondral scaffold (CartiHeal Agili‐C implant [Smith & Nephew, Andover, MA]) is designed to support simultaneous restoration of subchondral bone and overlying cartilage through host cell migration and matrix deposition but has not yet been shown to be suitable for arthroscopic implantation.^11^ The all‐arthroscopic technique described is technically demanding and not suitable for all patients because of anatomic constraints, patellofemoral mechanics, lesion characteristics, and the requirement for strict perpendicular access.

## SURGICAL TECHNIQUE

The described technique is a modification of the established technique via isolated open osteochondral implantation or as part of a larger surgical intervention.^11^, ^12^, ^13^ Indications are similar to those of open implantation, with additional patient‐specific considerations required to ensure safe arthroscopic execution.^8^, ^9^, ^14^

### Patient Positioning and Diagnostic Arthroscopy

The patient is positioned supine with the foot of the bed dropped to allow unrestricted motion of the knee. A proximal thigh holder is applied to permit controlled knee flexion and extension throughout the procedure. Standard anteromedial and anterolateral portals are used to perform diagnostic arthroscopy, evaluate intra‐articular pathology, and address concomitant conditions prior to cartilage restoration.

The trochlear defect is assessed for size, containment, geometry, and relation to patellar tracking. At this stage, a determination is made regarding the feasibility of an all‐arthroscopic implantation based on lesion characteristics and the ability to maintain perpendicular access for accurate socket preparation and press‐fit implantation (Table 1).

**TABLE 1:** Patient/Injury Requirements and Contraindications to Procedure

**Patient Presentation/Diagnostic Requirements**	**Contraindications**
• Symptomatic focal trochlear chondral or osteochondral defects	• Diffuse patellofemoral osteoarthritis
• Full‐thickness cartilage loss (International Cartilage Regeneration and Joint Preservation Society grade III‐IV) with or without subchondral bone involvement	• Uncorrected maltracking, instability, or severe trochlear dysplasia (Dejour classification C or D)
• Lesion size typically 1‐4 cm^2^	• Inflammatory arthropathy or systemic cartilage disease
• Contained defects with stable, vertical surrounding cartilage shoulders	• Inability to comply with postoperative rehabilitation
• Neutral or correctable patellofemoral mechanics	• Active infection or prior septic arthritis of the knee

**Relative Indications (Select Patients)**	**Relative Contraindications (Select Patients)**
• Focal bipolar patellofemoral disease with dominant trochlear‐sided pathology and preserved patellar cartilage	• Lesion size >4‐5 cm^2^ requiring multiple contiguous implants
• Young to middle‐aged, active patients seeking joint preservation	• Advanced obesity (body mass index >35‐40) because of increased patellofemoral load
• Patients for whom staged procedures (i.e., matrix‐induced autologous chondrocyte implantation) or donor‐site morbidity (i.e., osteochondral autograft transfer) are undesirable	• Untreated coronal or rotational malalignment affecting patellofemoral mechanics
	• Poor bone quality compromising press‐fit fixation

*Note*: General and technique‐specific indications, injury requirements, and contraindications to the all‐arthroscopic osteochondral implantation technique.

The foot is positioned on a Mayo stand to optimize knee flexion and extension control. For the all‐arthroscopic technique, the surgeon palpates and marks the anatomic borders of the patellar tendon position to avoid impingement on or disruption of the tendon and patella before committing to portal placement. An 18‐gauge spinal needle is used to confirm portal trajectory and required knee flexion while avoiding patellar tendon violation (Figure 1A‐C).

**FIGURE 1:**
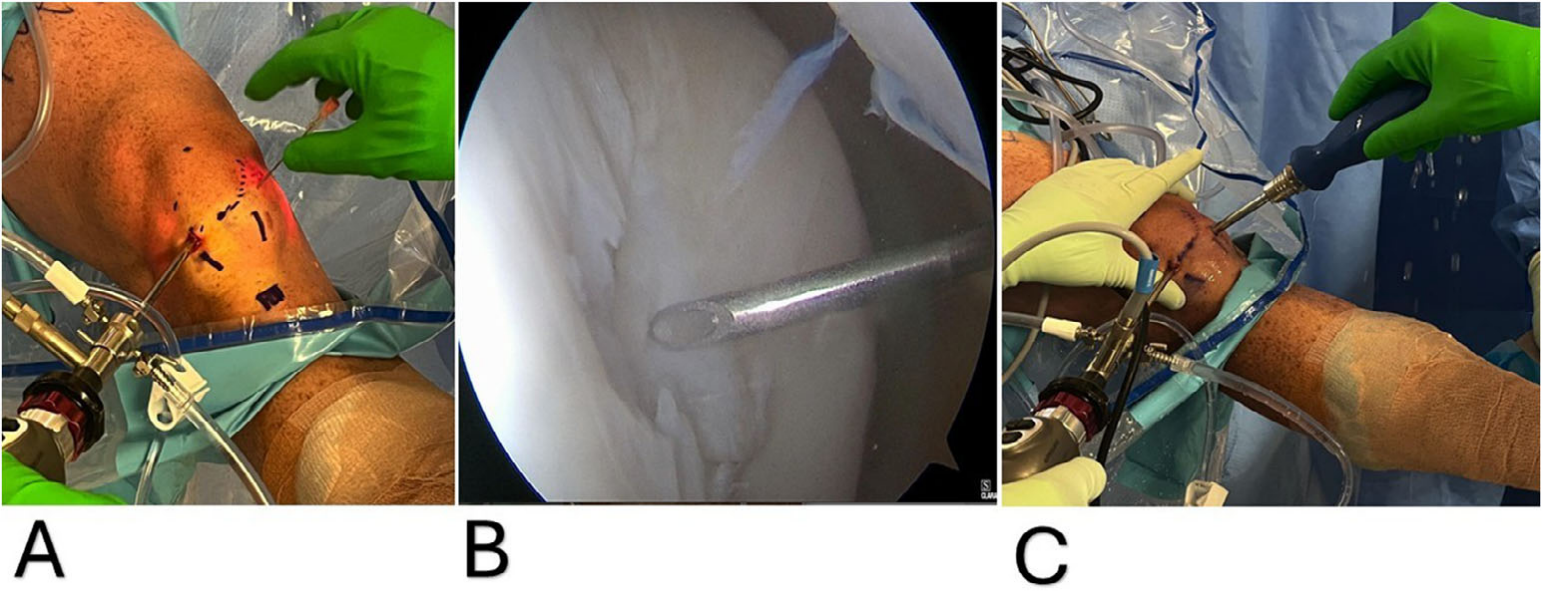
Portal placement, alignment, and creation. (A) A spinal needle is inserted perpendicularly to serve as a guide for portal placement of the right knee. (B) The arthroscope is positioned in the anterolateral portal to provide vision of the anteromedial portal through which the needle is positioned to align with the center of the cartilage defect. (C) The needle is removed, and the final working anteromedial port site is created based on the needle insertion point and trajectory.

Prior to portal establishment, the instrumentation is reviewed to ensure adequate portal sizing and a consistent trajectory throughout. Maintenance of a clear working corridor after portal placement is paramount to minimizing soft‐tissue disruption and facilitating controlled, reproducible instrumentation delivery (Video 1).

### Portal Planning and Lesion Preparation

Once the lesion is deemed amenable to arthroscopic implantation, an 18‐gauge 5‐inch spinal needle is used to plan the incisional trajectory. When a transpatellar tendon approach is necessary, fibers are split longitudinally to minimize iatrogenic injury and preserve tendon integrity.

The skin, subcutaneous tissue, fascia, and joint capsule are incised along the planned trajectory. Meticulous blunt dissection is then performed to avoid inadvertent chondral injury. A straight hemostat is used to spread the portal, followed by insertion of a switching stick. Sequential 7‐ and 8‐mm arthroscopic dilators (Arthrex, Naples, FL) are then introduced to enlarge the working channel. The portal must allow for a consistent trajectory and strict perpendicularity to the defect to facilitate controlled instrumentation delivery.

The lesion is debrided to stable vertical cartilage margins. The CartiHeal Agili‐C implant sizing and mapping device (Smith & Nephew) is used to plan the number, size, and spatial orientation of implants, ensuring a minimum 5‐mm bone bridge between sockets if using multiple implants. At least 180° of circumferential cartilage containment, preferably ≥270°, is required to optimize press‐fit fixation and biologic environment for integration.

### Socket Preparation

A perpendicular alignment guide is positioned flush against the articular surface and centered over the defect. The threaded guide pin is advanced until the black laser reference line is reached (Figure 2A‐C). The alignment guide is removed, and a drill bit with drill sleeve is advanced over the guide pin and drilled to its positive stop level (Figure 3A‐C). A cannulated hand reamer is then advanced over the guide pin and rotated in a clockwise fashion until burial of the black laser reference line, indicating appropriate depth, is confirmed via arthroscopic visualization (Figure 4A‐D). A proud laser line suggests insufficient depth and may predispose to proud implant positioning and subsequent failure.

**FIGURE 2:**
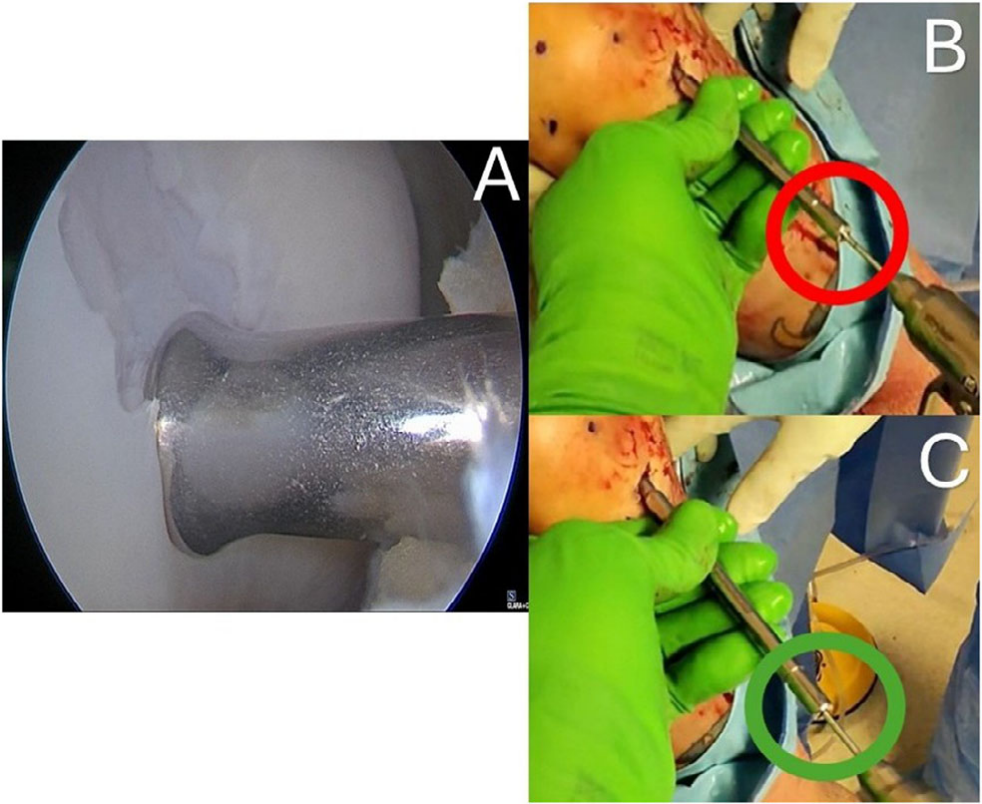
Alignment and K‐wire insertion. Orientation: laterality—right, arthroscopic port—anterolateral, and working port—anteromedial. (A) The specialized alignment guide is introduced through the anteromedial port and seated flush against the articular surface on the planned site for treatment. (B) The threaded K‐wire is advanced through the alignment guide using a drill with attention paid to the black laser line notated by the red circle. (C) The K‐wire insertion is completed at sufficient depth once the black laser line is no longer visible at the level of the guide (green circle).

**FIGURE 3:**
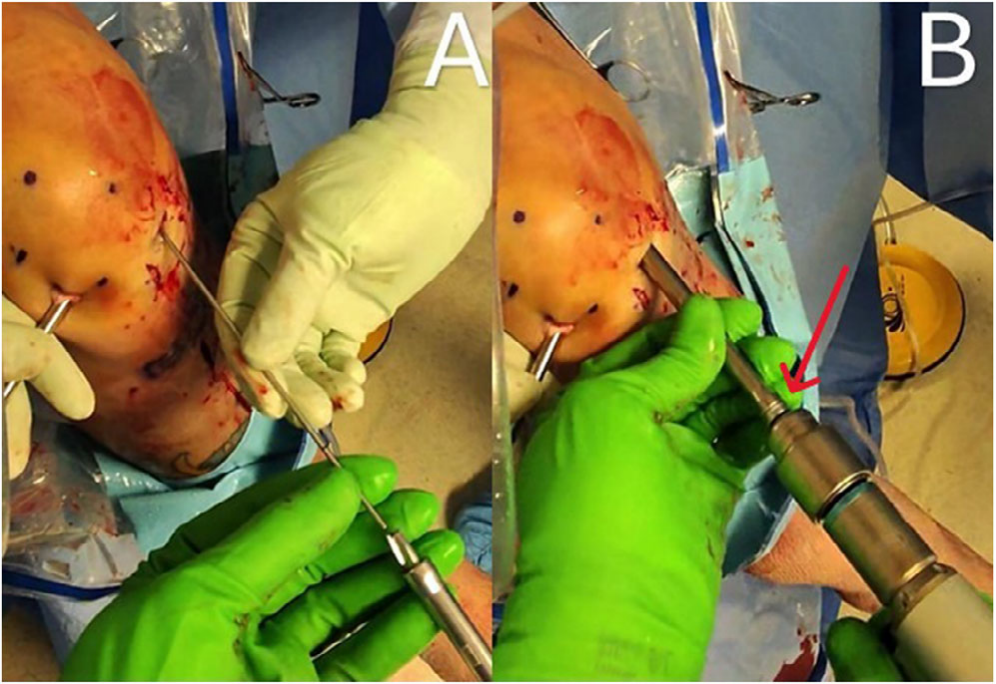
Socket drilling. Orientation: laterality—right, arthroscopic port—anterolateral, and working port—anteromedial. (A) The drill bit and drill sleeve are placed over the K‐wire in the anteromedial port. (B) With the drill sleeve seated flush on the articular surface, the drill is advanced to its positive stop on the guide (red arrow).

**FIGURE 4:**
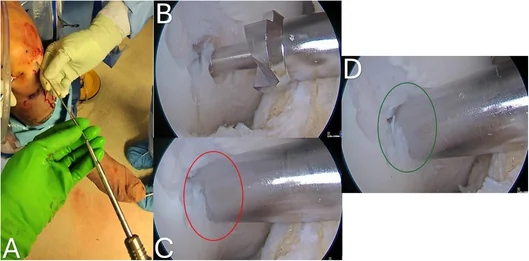
Socket reaming. Orientation: laterality—right, arthroscopic port—anterolateral, and working port—anteromedial. (A) The hand reamer is placed over the K‐wire in the anteromedial port. (B) The reamer is turned by hand in clockwise fashion. (C) The black laser line (red circle) is used to identify appropriate socket depth. (D) The black laser line must be buried below the articular surface circumferentially (green circle).

A fluted hand reamer is used to shape and refine socket geometry, achieving at least 3 nonresisted circumferential rotations, with frequent debris removal to maintain accuracy (Figure 5A‐D). The threaded guide pin is then removed. A cartilage beveling cutter is then used to contour the native cartilage margins (Figure 6A,B), facilitating chondrocyte migration and matrix deposition over the implant surface. The socket is inspected and cleared of debris with a motorized shaver prior to implantation.

**FIGURE 5:**
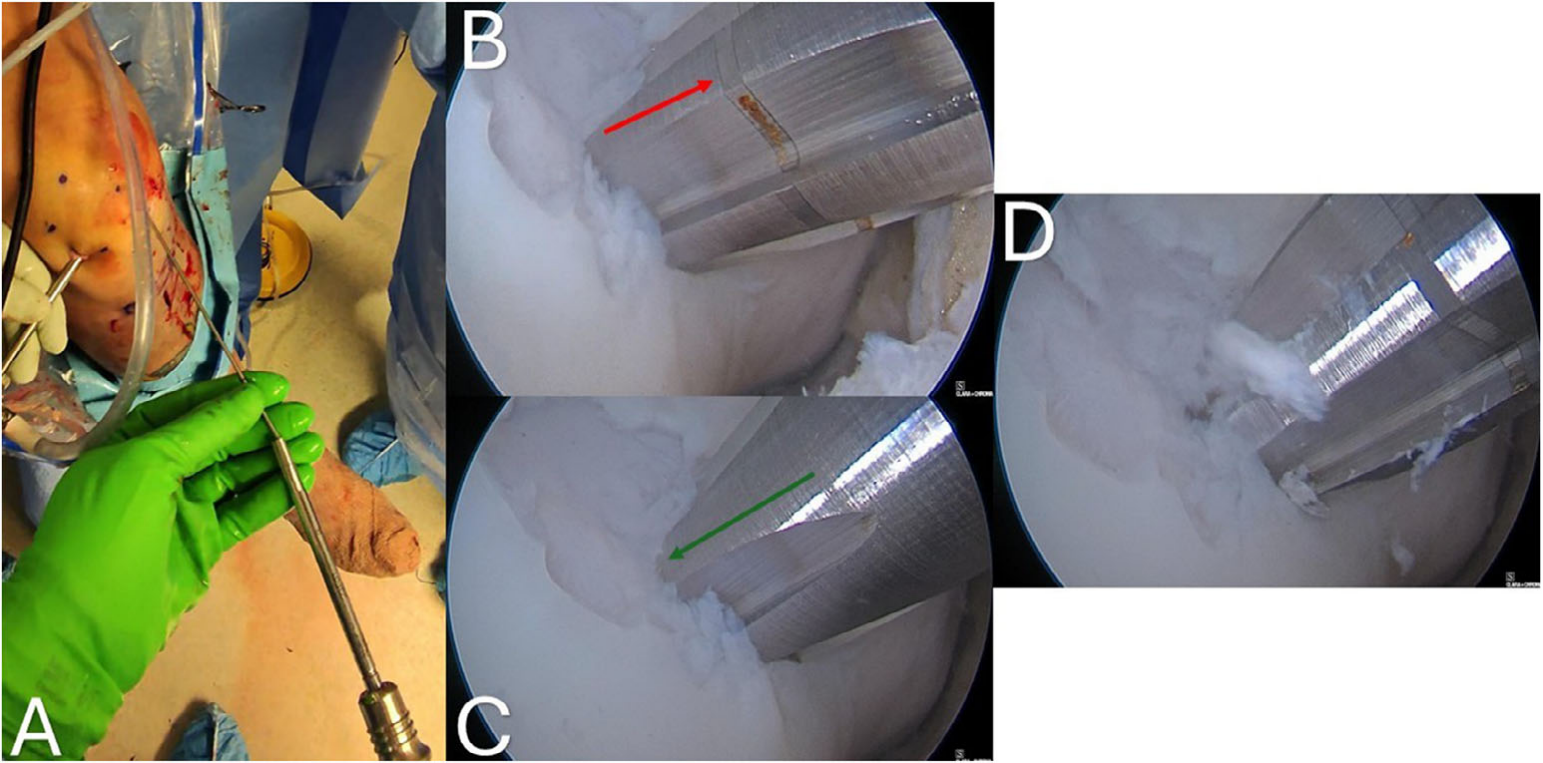
Socket shaping. Orientation: laterality—right, arthroscopic port—anterolateral, and working port—anteromedial. (A) The shaper is placed over the K‐wire in the anteromedial port. (B) The shaper is manually rotated in a clockwise fashion with care being paid to the black laser line (red arrow). (C) The shaper is advanced until the black laser line is buried (green arrow) and freely spinning with 2‐finger gentle force. (D) The flutes are cleared of debris in between sequential rotations.

**FIGURE 6:**
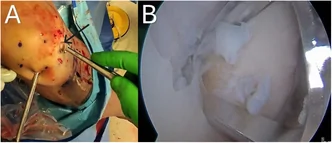
Socket beveling. Orientation: laterality—right, arthroscopic port—anterolateral, and working port, anteromedial. (A) The cartilage cutter device is inserted into the existing anteromedial portal (black arrow). (B) The cutter is turned by hand to trim and bevel the adjacent cartilage.

### Implant Delivery and Press‐Fit Fixation

A syringe‐based delivery device is fashioned using a sterile 3‐mL syringe on the back table (Figure 7). The plunger is withdrawn from the syringe; the black rubber seal gasket is removed and discarded to reduce the risk of intra‐articular fragmentation, leaving the clear plunger for controlled implant advancement (Figure 7B,C). The tip is sharply transected at the 0.1‐mL marking to create a smooth edge and conduit that sits flush over the prepared socket (Figure 7D,E).

**FIGURE 7:**
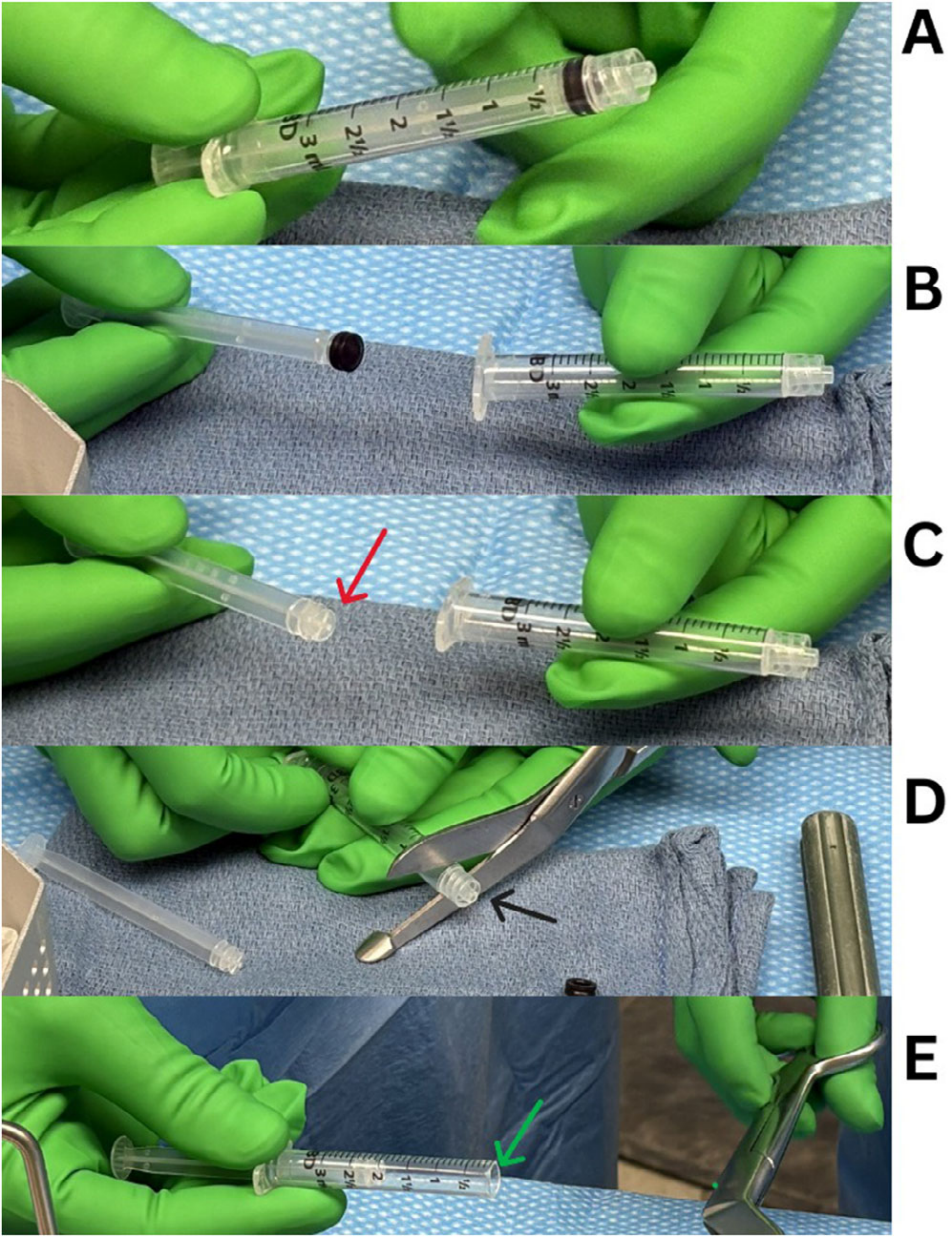
Modified syringe delivery device. (A) A sterile, standard 3‐mL Luer‐lock syringe is procured at the surgical table. (B) The plunger is withdrawn to separate the barrel and plunger components. (C) The black rubber seal gasket is removed from the plunger to leave only the clear plastic components (red arrow). (D) The syringe barrel is cut just behind the Luer‐lock hub assembly (black arrow) at the 0.1‐mL mark to create a wider opening. (E) The plunger is reintroduced to the syringe barrel to form a modified delivery apparatus with an open end (green arrow) to allow for scaffold insertion.

The syringe is seated flush over the prepared socket and held in position by an assistant while arthroscopic fluid inflow remains on and the surgeon changes gloves prior to handling the CartiHeal Agili‐C implant (Smith & Nephew). Arthroscopic inflow is temporarily discontinued, the plunger is withdrawn, and the implant is placed into the syringe barrel with the porous surface oriented toward the surgeon. Inflow is resumed, and under direct visualization, the implant is advanced into the socket using gentle axial pressure while maintaining perpendicular alignment (Figure 8A).

**FIGURE 8:**
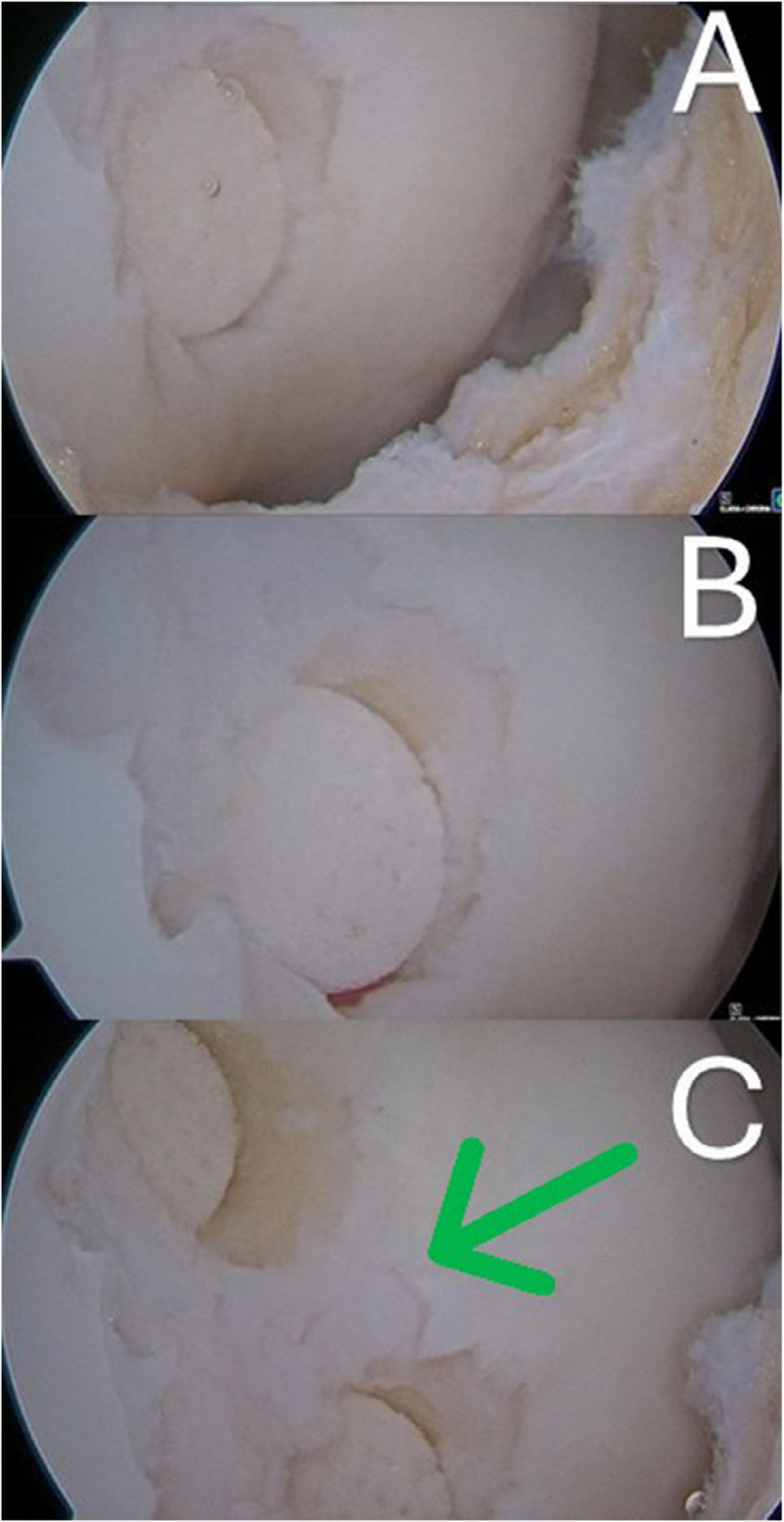
Implant positioning. Orientation: laterality—right. (A) Anterolateral view of the scaffold implant in provisional press‐fit position after placement with the modified syringe device. (B) Anterolateral view of the final scaffold implant placement with a press‐fit, stable, recessed position relative to surrounding articular cartilage. (C) Anterolateral view after implantation of multiple scaffold implants with bone bridge of ≥5 mm (green arrow) separating the implants.

Once provisional fixation is achieved, the syringe is removed, and the specialized CartiHeal tamp device (Smith & Nephew) is used to apply continuous axial pressure, seating the implant approximately 2 mm recessed relative to the surrounding cartilage. Final position and stability are confirmed arthroscopically (Figure 8B).

### Final Assessment and Closure

The knee is taken through a full range of motion (ROM) to assess implant stability and patellofemoral tracking. The implant is inspected for motion or edge incongruity. Additional implants are placed as needed, maintaining adequate bone bridges of at least 5 mm (Figure 8C). The joint is irrigated, portals are closed in standard fashion, and the knee is placed in a postop ROM brace.

### Postoperative Rehabilitation

Postoperative rehabilitation is similar to the course that the patient would complete if the implant were to be placed via mini‐arthrotomy. During weeks 0 to 6, the patient may bear weight as tolerated with their brace locked in extension while avoiding deep flexion and high patellofemoral loads with a goal to achieve 0° to 90° of ROM. From weeks 6 to 12, the patient is to complete progressive closed‐chain strength and ROM exercises to achieve a normal gait. From months 3 to 6, the patient will participate in proprioceptive training and functional progression to achieve impact clearance at 6 months. Final sport‐specific activity clearance is expected between 9 and 12 months.

## DISCUSSION

The all‐arthroscopic trochlear osteochondral scaffold implantation provides a single‐stage treatment option for focal trochlear defects while avoiding the morbidity of open exposure. Compared with staged procedures, autograft techniques, or osteochondral allograft transplantation, this approach eliminates donor‐site morbidity, secondary procedures, and graft‐related logistical challenges (Table 2).

**TABLE 2:** Advantages and Disadvantages of the All‐Arthroscopic Trochlear Osteochondral Scaffold Matrix Implantation

**Advantages**	**Disadvantages**
Single‐stage procedure	Technically demanding with a steep learning curve
Minimally invasive approach with reduced soft‐tissue disruption	Requires strict perpendicular access that may not be achievable in all patients
Avoids donor‐site morbidity associated with osteochondral autograft harvest	Limited to well‐contained focal defects
Eliminates need for staged cartilage harvest and reimplantation	Not appropriate for diffuse patellofemoral osteoarthritis
Avoids logistical constraints related to osteochondral allograft availability and sizing	Dependent on precise portal trajectory and instrumentation control
Preserves future cartilage restoration and arthroplasty options	May require intraoperative conversion to an open approach
Allows restoration of both subchondral bone and overlying cartilage	Limited long‐term outcome data for arthroscopic trochlear application
	The absence of a purpose‐built instrument requires creation of a custom tool by hand

The trochlear groove offers a favorable biomechanical environment for biphasic osteochondral scaffolds because of distributed contact pressures and reduced edge loading compared with femoral condylar lesions. When the osteochondral scaffold is implanted with intact circumferential bone support and appropriate cartilage containment, it allows restoration of both the subchondral bone and overlying cartilage in a single operative setting. However, this technique is technically demanding and is not appropriate for all patients. Successful execution requires the ability to achieve and maintain strict perpendicular access throughout drilling and implantation; inability to do so should prompt consideration of conversion to an open approach (Table 3). Limitations of this technique include a steep learning curve, dependence on precise portal trajectory, the need to fashion an improvised delivery device, and restriction to contained focal defects without advanced patellofemoral degeneration or uncorrected malalignment. Careful adherence to indications and intraoperative decision‐making is essential to optimize outcomes and avoid implant failure.

**TABLE 3:** Pearls and Pitfalls of the All‐Arthroscopic Trochlear Osteochondral Scaffold Matrix Implantation

**Surgical Step**	**Pitfalls**	**Pearls**
Diagnostic arthroscopy	Failure to correctly characterize the lesion (e.g., uncontained or diffuse) Inability to maintain visualization (fluid and soft‐tissue management) Unrecognized and untreated patellofemoral instability/malalignment	Confirmation of stable patellofemoral tracking and ability to maintain strict perpendicularity prior to committing to arthroscopic implantation
Portal planning and lesion access	Excessive patellar tendon violation or off‐axis portal trajectory	Use spinal needle localization to optimize trajectory while minimizing patellar tendon disruption
Lesion preparation and implant mapping	Inadequate lesion containment or improper implant spacing leading to convergence	Place adjacent to healthy cartilage >180°‐270° Ensure intact circumferential walls and maintain ≥4‐5 mm spacing between implants
Guide pin placement	Off‐center or oblique pin placement compromising socket geometry	Central pin placement dictates accurate reaming and press‐fit fixation
Socket preparation and reaming	Incomplete depth preparation or residual debris leading to proud implantation	Confirm burial of depth reference marker and clear debris between reaming passes
Cartilage edge preparation	Residual cartilage or soft tissue impeding press‐fit fixation	Circumferential cartilage beveling to achieve unobstructed host bone contact
Implant delivery	Loss of perpendicular alignment or soft‐tissue drag during insertion	Syringe‐based delivery maintains controlled trajectory and visualization
Press‐fit implantation	Proud or unstable implant positioning increasing contact pressures	Seat implant flush to slightly recessed relative to surrounding cartilage
Final assessment and closure	Failure to assess dynamic patellofemoral tracking	Perform full range‐of‐motion testing to confirm implant stability

*Note*: Step‐by‐step pearls and pitfalls of the all‐arthroscopic osteochondral implantation technique.

For select patients, the all‐arthroscopic trochlear osteochondral scaffold implantation technique is a minimally invasive alternative to open techniques that may reduce soft‐tissue morbidity and preserve future surgical options. Although promising, the technique is not appropriate for all patients and lacks long‐term outcome data. Ongoing monitoring and further studies are necessary to define safety, efficacy, and broader applicability.

## DECLARATION OF GENERATIVE AI AND AI‐ASSISTED TECHNOLOGIES IN THE WRITING PROCESS

During the preparation of this manuscript, the authors used ChatGPT (OpenAI) to refine grammar, clarity, and brevity. After using this tool/service, the authors reviewed and edited the content as needed and take full responsibility for the content of the published article.

## DISCLOSURES

The authors (M.V., J.H.) declare the following financial interests/personal relationships which may be considered as potential competing interests: M.V. reports a relationship with Arthrex, Smith & Nephew, Johnson & Johnson MedTech, and Pacira BioSciences that includes consulting or advisory. J.H. reports a relationship with Pacira BioSciences that includes employment and equity or stocks.
